# ASO Author Reflections: The Effect of Local Adjuvants on Cortical Bone Following Intralesional Curettage of Bone Tumors

**DOI:** 10.1245/s10434-024-15473-9

**Published:** 2024-06-01

**Authors:** Vasileios Apostolopoulos, Petr Boháč, Petr Marcián, Iva Staniczková Zambo, Lukáš Pazourek, Michal Mahdal, Jakub Neradil, Tomáš Návrat, Tomáš Tomáš

**Affiliations:** 1 First Department of Orthopaedic Surgery, St. Anne’s University Hospital and Faculty of Medicine, Masaryk University, Brno, Czech Republic; 2 Institute of Solid Mechanics, Mechatronics and Biomechanics, Faculty of Mechanical Engineering, University of Technology, Brno, Czech Republic; 3 First Department of Pathology, St. Anne’s University Hospital and Faculty of Medicine, Masaryk University, Brno, Czech Republic; 4 Laboratory of Tumor Biology, Department of Experimental Biology, Faculty of Science, Masaryk University, Brno, Czech Republic; 5 International Clinical Research Center, St. Anne’s University Hospital, Brno, Czech Republic

## Past

The irregular shape of the tumor cavity in certain types of tumors poses a significant challenge in completely removing both microscopic and macroscopic tumor residues. Consequently, higher rates of local recurrence following tumor curettage have been recorded.^1^ To lower those rates, curettage should be supplemented with other local adjuvants. The objective of local adjuvant therapy is to eradicate any remaining viable tumor cells, ensuring complete removal while preserving the integrity of nearby healthy bone and surrounding soft tissue.^2^ Various treatment combinations are employed upon curettage for both benign and locally aggressive bone tumors. Despite the array of available treatment modalities, notable complication rates persisted, with postoperative fractures emerging as the most prevalent complication during follow-up periods.^3^ Thus far, no studies have comprehensively evaluated the effect of topical adjuvants on cortical bone after intralesional bone curettage by measuring density and hardness.

## Present

Currently, there is no consensus regarding the choice of local adjuvant therapy for tumor treatment. Consequently, the selection of local adjuvants varies among surgeons, with the most common options including phenol, electrocautery, argon beam coagulation, and liquid nitrogen, as well as other substances. Some authors also utilize polymethyl methacrylate (PMMA) and hydrogen peroxide. Each adjuvant therapy offers potential advantages and associated risks.^4^ Existing studies have analyzed the depth of necrosis induced by local adjuvants and compared the complication rates accordingly.^5^ The present companion article provides a comprehensive analysis of the impact of local adjuvants on porcine cortical bone, assessing bone density (via micro-computed tomography [CT] and hydroxyapatite calibration phantom), bone hardness, and depth of necrosis.^6^ Notably, phenolization and liquid nitrogen application were found to decrease bone hardness, with bone density being marginally affected; however, liquid nitrogen application led to extensive necrosis depth. Conversely, argon beam coagulation and electrocautery demonstrated adequate penetration without adversely affecting bone hardness.^6^ However, the optimal treatment modality remains unsettled. Ongoing research indicates that the effectiveness of local adjuvant therapy depends on the specific type of tumor being treated.^4^

## Future

Further clinical investigation is required to validate the results of this experimental study. The authors suggest that forthcoming research efforts should focus on developing targeted chemical local adjuvants to advance precision treatment modalities. Investigating novel low-molecular-weight inhibitors or alternative forms of localized targeted therapy has the potential to decrease local recurrence rates while safeguarding the integrity of neighboring healthy bone tissue. By evolving specific molecular targets and refining therapeutic approaches at the local level, there is a promising opportunity to enhance treatment efficacy and minimize adverse effects on surrounding structures.
